# High-resolution African population projections from radiative forcing and socio-economic models, 2000 to 2100

**DOI:** 10.1038/sdata.2016.130

**Published:** 2017-01-17

**Authors:** Niklas Boke-Olén, Abdulhakim M. Abdi, Ola Hall, Veiko Lehsten

**Affiliations:** 1 Department of Physical Geography and Ecosystem Science, Lund University, Sölvegatan 12, SE-223 62 Lund, Sweden; 2 Department of Human Geography, Lund University, Sölvegatan 10, SE-223 62 Lund, Sweden; 3 Swiss Federal Institute for Forest, Snow and Landscape research (WSL), Zürcherstr. 11, CH-8903 Birmensdorf, Switzerland

**Keywords:** Climate change, Environmental chemistry, Geography, Socioeconomic scenarios

## Abstract

For its fifth assessment report, the Intergovernmental Panel on Climate Change divided future scenario projections (2005–2100) into two groups: Socio-Economic Pathways (SSPs) and Representative Concentration Pathways (RCPs). Each SSP has country-level urban and rural population projections, while the RCPs are based on radiative forcing caused by greenhouse gases, aerosols and associated land-use change. In order for these projections to be applicable in earth system models, SSP and RCP population projections must be at the same spatial scale. Thus, a gridded population dataset that takes into account both RCP-based urban fractions and SSP-based population projection is needed. To support this need, an annual (2000–2100) high resolution (approximately 1km at the equator) gridded population dataset conforming to both RCPs (urban land use) and SSPs (population) country level scenario data were created.

## Background & Summary

The size and future trend of the human population on Earth has been a topic of scientific enquiry at least since Thomas R. Malthus wrote *An Essay on the Principle of Population* in 1798. Presently, the Population Division of the United Nations (UN) is one of several agencies or institutions that publish global population projections on a country-by-country basis at predefined intervals. The UN’s projections are published in a biennial report called the World Population Prospects (WPP) with the 2015 revision projecting a remarkable 270% increase in the African population between 2015 and 2100 (ref. 1). This rapid population growth has a number of potentially associated effects that could fundamentally alter the continent’s landscape. These include high rates of urbanization leading to unplanned expansion of cities, the spread of informal settlements that lack basic services and degradation of natural resources as a result of over-exploitation to meet rising food demand^2,3^. Furthermore, a projected 4 °C global warming by 2100 could have severe negative impacts on the rain-fed agro-ecosystems that are a main source of livelihood and nutrition in sub-Saharan Africa (SSA)^3,4^. Altogether, this will lead to a reallocation of population from rural to urban areas, and create strong incentives for landscape transformation.

Population data has traditionally taken the form of estimates per administrative unit per unit time. The administrative units are often in the form of regional, national, or sub-national counts with temporal units at best at annual intervals but more commonly 5–10 years. From an analytical perspective this is troublesome. Since administrative units vary in size, shape, and usually are arbitrarily defined any study that uses administrative borders will obtain a very heterogeneous set of observations. Since the 1990s, there have been growing attempts to disaggregate population data into spatially explicit estimates distributed across a regular grid. Tobler, *et al.*^5^ produced a smoothed continuous map-grid of total population at a global scale for the year 1994. This work was later labeled as the first version of the Gridded Population of the World (GPWv1) by the Center for International Earth Science Information Network (CIESIN). The fourth, and latest, version, GPWv4, was published in 2015 (ref. 6) and includes population data at 5-year intervals between 2000 and 2020. As part of the AfriPop project, Linard *et al.*^7^ built a unique spatial database that combined census data across the continent with satellite-imagery and land cover data in a dasymetric model. The AfriPop project was transformed into an open access archive and re-labeled WorldPop. Within this new framework, Sorichetta *et al.*^8^ developed a gridded population distribution dataset for the Caribbean and Latin America for 2000, 2015 and 2020. However, due to the coarse temporal resolution and limited time period available for these datasets none of them can be completely used as an accompanying dataset for analyses using projected 21th century data within Intergovernmental Panel on Climate Change (IPCC) Fifth Assessment Report (AR5) framework which is based on gridded climate projection data.

Here, we present a 30 arc-second, gridded population distribution projection for every year between 2000 and 2100 for the African continent conforming to both the Representative Concentration Pathways (RCPs) urban fractions^9^ and Shared Socio-Economic Pathways (SSPs) population (major characteristics presented in Table 1). To our knowledge the combination of RCPs and SSPs scenarios for gridded population projections is something that has not been done before. The RCPs and SSPs supersedes the Special Report on Emissions Scenarios (SRES) and were adopted by the IPCC for its AR5 (refs 3,^10^). Unlike the SRES, the four RCPs are not based on socio-economic scenarios but on radiative forcing and the simulated influences of land use, greenhouse gas and aerosol emissions. Therefore, a set of five SSPs were used in conjunction with the RCPs to develop future country level population distribution scenarios^11^. The development of society and the natural environment in SSPs are not explicitly taking climate change or the implementation of climate policies at the global scale during the 21st century into account. Each SSP scenario is adjoined with a population projection and a proportion of the country population living in urban areas. The presented gridded population dataset can be useful when performing future simulations dependent on gridded RCP land use and climate data, for example carbon flux studies or assessments of supply and demand of food.

## Methods

The method can be summarized as a distribution of country level SSP urban and rural population projections onto a 30 arc seconds grid conforming to the urban fraction grid at 30 arc minutes. For this we used the African population for year 2000 at 30 arc-second spatial resolution from the WorldPop Project^12^ as a starting dataset. Distance to road and to population centers of gravity were used to allow each pixel to be ranked uniquely into urban or no-urban (see below). A complete list of the included data sources can be found in Table 2.

### SSP-RCP coupling in the baseline year 2000

The population distribution for Africa was modeled to follow the RCP-specific urban fraction dataset^13^ further described in Hurtt *et al.*^9^ and the country-specific SSP population and urban fraction scenarios from the SSP database^14^. The urban fraction data is provided at a spatial resolution of 30 arc-minutes (0.5 degrees or approximately 50 kilometers at the equator) and represents annual projected global land use and land cover patterns until the year 2100. It is developed with a Global Land-use Model (GLM)^15^ which estimates future land use transitions and patterns within each 30 arc-minute grid cell using an accounting based method by considering a range of parameters (i.e. spatial patterns, residency time, and land conversions)^9^. The five SSPs and four RCPs produce a set of 20 SSP-RCP scenario combinations that deliver a reasonable basis for future scenarios. However, some combination (e.g., SSP1-RCP8.5) are very unlikely to occur in the future^16^ (p<0.01). Consequently, we used the 15 most probable SSP-RCP combinations according to Engström *et al.*^17^.

**Figure 1:**
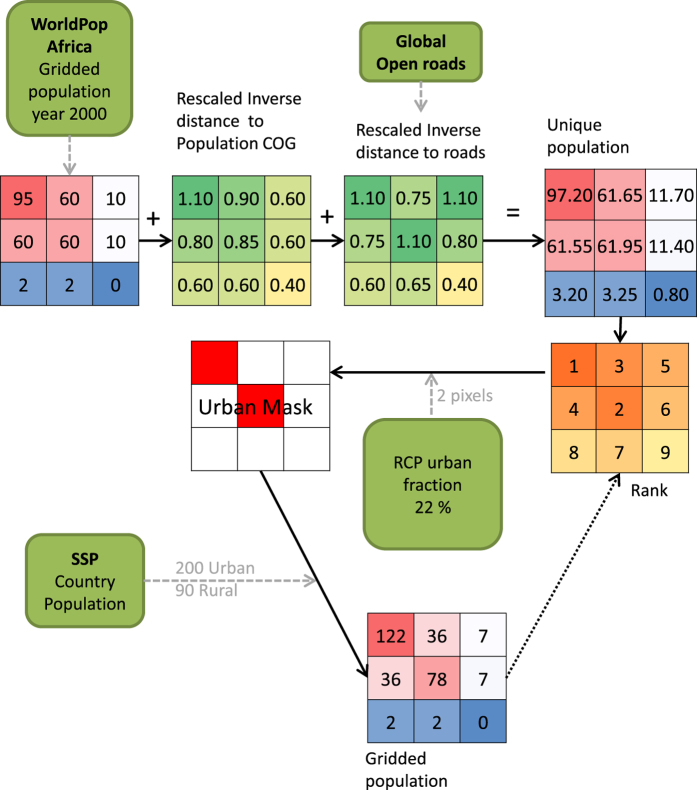
Flowchart and example of the method used to distribute the population. Example made with artificial numbers for visualization purposes. For simplicity the smaller grid cells each corresponds to one 30 arc second pixel and the full grid (9×9) represent one urban fraction grid cell (0.5 degree) and the full grid 9x9 corresponds to one country. The example highlights the need to add inverse distance to population center of gravity (COG) and inverse distance to roads to the population data to be able to uniquely rank the pixels. The country SSP population is distributed for rural and urban separately based on the urban mask and the gridded population from the year before or unique population for the first year. The green boxes with rounded corners indicate input data. Inverse distance to center of gravity and inverse distance to roads are for the small example not rescaled to the same range as done when processing the dataset.

The gridded population dataset for Africa for the year 2000 (ver. 3.020) from the WorldPop Project^12^ was used as the starting point. The estimates of total number of people per grid cell across Africa are adjusted to match UN population division estimates. The national administrative boundaries dataset from GRUMPv1 (refs 18,^19^) were used to remove water bodies from the WorldPop data. This was done by converting the national boundaries to raster using ArcGIS 10 raster to polygon tool with 30 arc-seconds as the output resolution. The created raster was reclassified to include only ones and zeros for land and water bodies, respectively. Finally, it was matched to the spatial origin of WorldPop by using the resample (nearest neighbor) function within the raster package in R (R: A Language and Environment for Statistical Computing, https://www.r-project.org/). To classify each 30 arc-second pixel within each 0.5 degree pixel as urban or non-urban based on their population value the inverse distance to roads and inverse distance to center of gravity (COG) were each rescaled to lie between 1.0·10^−5^ and 1.1·10^−5^ and added to the initial dataset (Fig. 1). Distance to roads and distance to population centers (COG) were chosen since their effect on the population distribution within an area have been demonstrated repeatedly^8,20–22^. The distance to roads was calculated using Euclidian distance on the global roads open access data set^23^. The COG was calculated on the WorldPop dataset for each 0.5 degree urban fraction pixel using the COGravity tool included in the R (R: A Language and Environment for Statistical Computing, https://www.r-project.org/) package SDMTools (https://cran.r-project.org/web/packages/SDMTools/). These operations are done to be able to more accurately classify pixels as urban or non-urban based on their unique population value and also to ensure that urban growth will be favored in proximity to population centers or roads. Our procedure requires ranking the pixels as non-urban or urban based on their population values. Hence a maximum of two arc-second pixels within each 30 arc-minute urban fraction grid cell were allowed to have the exact same value. This was achieved by addition of the rescaled inverse distance to road and COG, the result of that is from now on referred to as unique population dataset.

### Population allotment per grid cell

The unique population dataset for year 2000 is used together with the RCP specific urban fraction for year 2000 to create the urban mask (Fig. 1). This is done by sorting the pixel values within each urban fraction 0.5 degree grid cell and selecting the highest ones as urban until the urban fraction value is fulfilled (rounding the number of urban pixels within each 0.5 degree grid cell to the nearest integer). This is repeated for all 0.5 degree grid cells. Subsequently, the urban mask is used on a per country basis to distribute the urban and rural SSP population data and assuring that the unique population relationship is still valid. This means, for example, that a pixel with an initial value twice as large as another pixel will still have the same ratio under the condition that they are both located in the same country and are both either urban or rural pixels. The gridded population of the previous year is used as input to create the urban mask of the next year and to distribute the population thus creating a loop (Fig. 1). This ensures that the unique population for year 2000 will only be used once to create the dataset for year 2001 and thereafter the population from the year before will be used as the unique data set (Fig. 1). This meaning that the population from the previous year will be used to create a new urban mask based on the RCP urban fraction for that year. Hence the spatial distribution of the urban areas is based on the yearly RCP urban fraction and the gridded population from the year before using the ranking technique as shown in Fig. 1.

**Figure 2:**
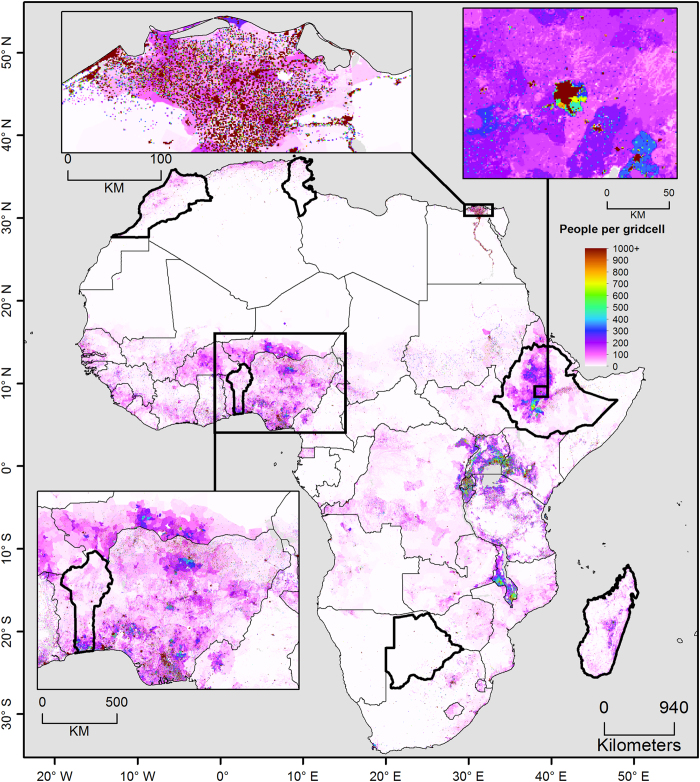
Map of year 2050 of the gridded population datasets for SSP 2 and RCP 4.5. Countries presented with bold borders are the countries used for a more in depth comparison (see technical validation section).

The population re-distributions are carried out for the 15 likely combinations of RCP and SSP scenarios from Engström *et al.*^17^ (Table 3). Hence, in total 15 different datasets with yearly population projections between 2000 and 2100 are created. An example of the population re-distribution for SSP 2 and RCP 4.5 for year 2050 can be seen in Fig. 2.

### Code availability

The gridded population datasets were created using R3.3.0 (R: A Language and Environment for Statistical Computing, https://www.r-project.org/) and the scripts can be found on GitHub (https://github.com/niklasbokeolen/african_population/).

## Data Records

The high resolution population projections for the RCP and SSP scenario combinations described here can be freely and publicly accessed at the DataGURU web site (Data citation 1) which also allows basic conversion/spatial and temporal cropping, thus enhancing the accessibility. The original data are stored as one geotiff (.tif) for each scenario combination and year with the datatype FLT4S and NA-value as -9999. They are in a longitude latitude projection with WGS84 as the datum and are created with the function writeRaster within the raster package (v2.5-2, https://cran.r-project.org/web/packages/raster/) in R 3.3.0 (R: A Language and Environment for Statistical Computing, https://www.r-project.org/).

To get an overview of the created dataset, parts of it can be explored with a shiny web application which provides an interactive visualization. It can be found in the below location:https://niklasbokeolen.shinyapps.io/Shiny_population/.

Note that the tool uses a re-projected version of the data to match the projection of the OpenStreetMap. For the official version of the gridded population dataset, the user is advised to download the original files via the DataGURU service which also provides the long term storage.

## Technical Validation

**Figure 3:**
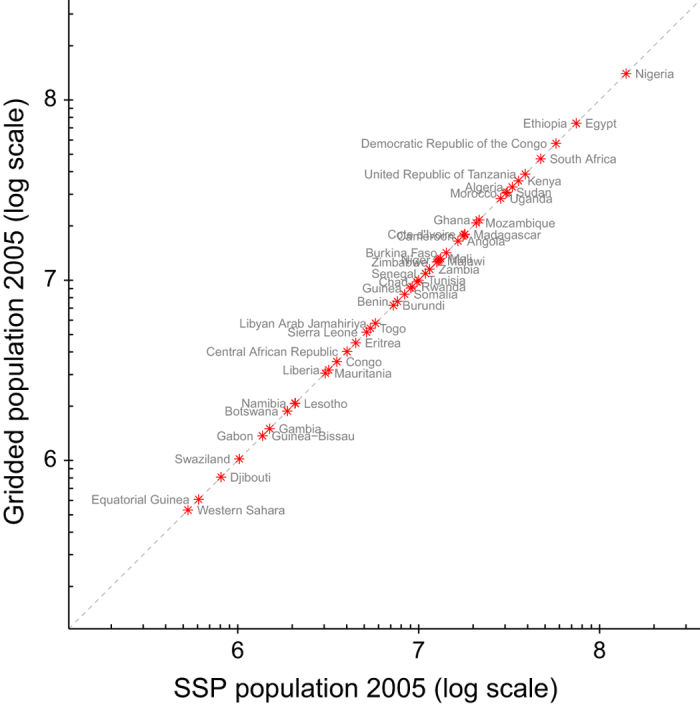
Country level validation of gridded dataset for year 2005. Gridded population aggregated to country totals and compared against SSP country population.

The technical validation of the dataset is performed by comparing the SSP 1 country populations with the gridded population dataset (for SSP1/RCP 4.5) aggregated to country levels. This is done for year 2005 since both SSP population projections and RCP land use projections do not deviate until after that year. Accordingly, we performed the technical validation on only one RCP–SSP combination. We show that all the population is accounted for and every country lies on the one to one line (Fig. 3) with a coefficient of determination (r^2^) of 1.0.

**Figure 4:**
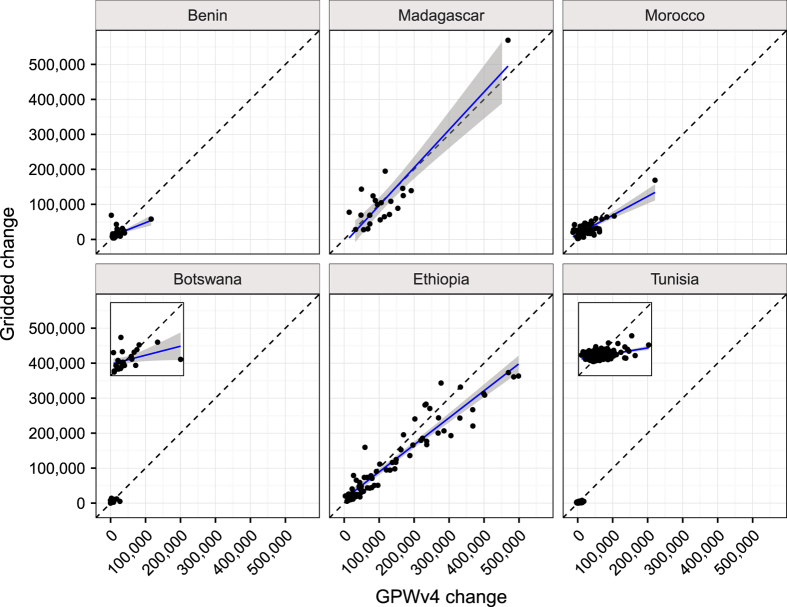
Validation result on administrative region at level 2. Plots show population change between year 2000 and 2005 for data set created in this paper (gridded change) and gridded population of the world v4 (GPWv4 change). A zoom of the Botswana and Tunisia data is included as an inset.

To further evaluate the created dataset we compared the 2000 to 2005 population change for the created gridded dataset with the change for UN (2015) adjusted Gridded Population of the World version 4 (GPWv4). This was done for a sample of six African countries (Benin, Madagascar, Morocco, Botswana, Ethiopia, and Tunisia) representing a wide variation in population density and spatial distribution. The population was aggregated to level 2 administrative regions within each country and only one SSP/RCP combination was used since the projections do not deviate until after year 2005. The result of the comparison to GPWv4 can be seen in Fig. 4. For most of the countries the change matches in general well but typically deviates more for a few of the level 2 administrative regions. A summary of the comparison to GPWv4 for the validation countries and year 2005 can be seen in Table 4 were it can be observed that the total distributed populations (SSP countries population) are not exactly the same as the population for GPWv4. However, the coefficient of determination is high (r^2^>0.8) for most of the countries and administrative levels indicating that the spatial pattern is captured between the regions. This indicates that the method is well suited to capture the spatially pattern. However, due to considerable differences in the total population between GPWv4 and the SSP population data we do not expect the change to match perfectly for all regions.

### Uncertainty

We used a deterministic method to produce the gridded population projections which means that the level of uncertainty in the created dataset is originating from uncertainty in the input data (Table 2). We would like to point out a possibly over-influence of roads in the created dataset. This is due to that WorldPop uses distance to roads and we further add the inverse distance to roads in order to create a unique population dataset as a starting point. However, since we rescale the distance to road to be between 1.0·10^−5^ and 1.1·10^−5^ we argue that this will only have an effect for pixels that were equal in the initial WorldPop dataset. Pixels (30 arc-seconds) with unequal population values in the initial dataset will have a very low probability of being affected by this small addition based on the distance to road.

## Additional information

**How to cite this article**: Boke-Olén, N. *et al.* High-resolution African population projections from radiative forcing and socio-economic models, 2000 to 2100. *Sci. Data* 4:160130 doi: 10.1038/sdata.2016.130 (2017).

**Publisher**’**s note**: Springer Nature remains neutral with regard to jurisdictional claims in published maps and institutional affiliations.

## Supplementary Material



## Figures and Tables

**Table 1:** Main characteristics of RCP and SSP assumptions.

**Pathway**	**Key characteristics**
RCP2.6	-Radiative forcing 2.6 W m^−2^ by 2100
	-Low GHG emissions
	-Medium-low air pollution
RCP4.5	-Radiative forcing 4.5 W m^−2^ by 2100
	-Very low baseline GHG emission with medium-low mitigation
	-Medium air pollution
RCP6.0	-Radiative forcing 6.0 W m^−2^ by 2100
	-Medium baseline GHG emission with high mitigation
	-Medium air pollution
RCP8.5	-Radiative forcing 8.5 W m^−2^ by 2100
	-High baseline GHG emission
	-Medium-High air pollution
SSP1	-Low population
	-High urbanization
	-High-Medium Economy
SSP2	-Medium population
	-Medium urbanization
	-Medium uneven economy
SSP3	-High population
	-Low urbanization
	-Slow economy
SSP4	-Medium-High population
	-High-Medium urbanization
	-Low-Medium economy
SSP5	-Low-Medium Population
	-High Urbanization
	-High Economy
RCP characteristics from Van Vuuren *et al.*^10^ and SSP characteristics from O’Neill *et al*.^24^ and Kc & Lutz^25^.	

**Table 2:** Input datasets used to grid future populations.

**Name**	**Spatial domain**	**Temporal domain**	**Type**	**Source**
SSP population scenarios	Country	2000–2100	Continuous	SSP Database^14^
RCP Urban fraction	0.5 degree	2000–2100	Raster	Chini *et al.*^13^
WorldPop Africa	30 arc-second	2000	Raster	http://www.worldpop.org/
Roads	Global	1980–2010	Polylines	gROADSv1^23^
Water bodies mask	Global	2000	Polygons	GRUMPv1:National-Administrative-Boundaries^18,19^
Country Borders	Global	2008	Polygons	http://www.thematicmapping.org/downloads/

**Table 3:** RCP–SSP probability matrix as described by Engström *et al.*^17^

	**RCP 2.6**	**RCP 4.5**	**RCP 6**	**RCP 8.5**
SSP 1	0.09	0.45	0.45	0.00
SSP 2	0.00	0.09	0.68	0.23
SSP 3	0.00	0.17	0.50	0.33
SSP 4	0.00	0.37	0.56	0.07
SSP 5	0.00	0.07	0.37	0.56

**Table 4:** Summary of gridded data (presented dataset) countries aggregated to administrative region level 1 and 2 for year 2005 and compared with GPWv4.

	**n**	**r**^**2**^	**RMSE**	**GPWv4 average population**	**Gridded average population**	**GPWv4 country population**	**SSP country population**
*Benin*						8,203,291	7,630,000
* Adm1*	12	0.89	104,369	674,354	631,009		
*Adm2*	76	0.81	38,391	106,477	99,633		
*Madagascar*						18,200,149	17,900,000
*Adm1*	6	0.91	635,232	3,028,272	2,976,479		
*Adm2*	22	0.86	407,449	825,892	811,767		
*Morocco*						29,920,484	30,400,000
*Adm1*	15	0.79	587,860	1,988,823	2,015,600		
*Adm2*	54	0.91	312,416	552,451	559,889		
*Botswana*						1,860,717	1,880,000
*Adm1*	9	0.98	26,917	205,694	208,228		
*Adm2*	25	0.52	47,608	74,050	74,962		
*Ethiopia*						76,536,708	74 300 000
*Adm1*	11	0.99	1,162,510	6,953,130	6 749 082		
*Adm2*	72	0.94	229,176	1,062,284	1,031,110		
*Tunisia*						9,767,114	10,000,000
*Adm1*	24	0.83	174,471	404,435	414,169		
*Adm2*	268	0.48	26,375	36,218	37,090		
SSP country population is the population from the SSP scenarios that the product is based on. The SSP population scenarios do not deviate until after year 2005. n indicates number of regions within each administrative level. r^2^ is the coefficient of determination and RMSE is the root mean square error between GPWv4 and gridded population, aggregated to administrative level one or two.							
